# Identifying Deprescribing Opportunities With Large Language Models in Older Adults: Retrospective Cohort Study

**DOI:** 10.2196/69504

**Published:** 2025-04-11

**Authors:** Vimig Socrates, Donald S Wright, Thomas Huang, Soraya Fereydooni, Christine Dien, Ling Chi, Jesse Albano, Brian Patterson, Naga Sasidhar Kanaparthy, Catherine X Wright, Andrew Loza, David Chartash, Mark Iscoe, Richard Andrew Taylor

**Affiliations:** 1 Department of Biomedical Informatics and Data Science School of Medicine Yale University New Haven, CT United States; 2 Program of Computational Biology and Bioinformatics Yale University New Haven, CT United States; 3 Department of Emergency Medicine School of Medicine Yale University New Haven, CT United States; 4 VA Connecticut Healthcare System US Department of Veterans Affairs West Haven, CT United States; 5 Department of Pharmacy Yale New Haven Hospital New Haven, CT United States; 6 BerbeeWalsh Department of Emergency Medicine University of Wisconsin–Madison Madison, WI United States; 7 Section of Cardiovascular Medicine Department of Internal Medicine Yale School of Medicine New Haven, CT United States; 8 School of Medicine University College Dublin Dublin Ireland; 9 Department of Biostatistics School of Public Health Yale University New Haven, CT United States

**Keywords:** deprescribing, large language models, geriatrics, potentially inappropriate medication list, emergency medicine, natural language processing, calibration

## Abstract

**Background:**

Polypharmacy, the concurrent use of multiple medications, is prevalent among older adults and associated with increased risks for adverse drug events including falls. Deprescribing, the systematic process of discontinuing potentially inappropriate medications, aims to mitigate these risks. However, the practical application of deprescribing criteria in emergency settings remains limited due to time constraints and criteria complexity.

**Objective:**

This study aims to evaluate the performance of a large language model (LLM)–based pipeline in identifying deprescribing opportunities for older emergency department (ED) patients with polypharmacy, using 3 different sets of criteria: Beers, Screening Tool of Older People’s Prescriptions, and Geriatric Emergency Medication Safety Recommendations. The study further evaluates LLM confidence calibration and its ability to improve recommendation performance.

**Methods:**

We conducted a retrospective cohort study of older adults presenting to an ED in a large academic medical center in the Northeast United States from January 2022 to March 2022. A random sample of 100 patients (712 total oral medications) was selected for detailed analysis. The LLM pipeline consisted of two steps: (1) filtering high-yield deprescribing criteria based on patients’ medication lists, and (2) applying these criteria using both structured and unstructured patient data to recommend deprescribing. Model performance was assessed by comparing model recommendations to those of trained medical students, with discrepancies adjudicated by board-certified ED physicians. Selective prediction, a method that allows a model to abstain from low-confidence predictions to improve overall reliability, was applied to assess the model’s confidence and decision-making thresholds.

**Results:**

The LLM was significantly more effective in identifying deprescribing criteria (positive predictive value: 0.83; negative predictive value: 0.93; McNemar test for paired proportions: χ^2^_1_=5.985; *P*=.02) relative to medical students, but showed limitations in making specific deprescribing recommendations (positive predictive value=0.47; negative predictive value=0.93). Adjudication revealed that while the model excelled at identifying when there was a deprescribing criterion related to one of the patient’s medications, it often struggled with determining whether that criterion applied to the specific case due to complex inclusion and exclusion criteria (54.5% of errors) and ambiguous clinical contexts (eg, missing information; 39.3% of errors). Selective prediction only marginally improved LLM performance due to poorly calibrated confidence estimates.

**Conclusions:**

This study highlights the potential of LLMs to support deprescribing decisions in the ED by effectively filtering relevant criteria. However, challenges remain in applying these criteria to complex clinical scenarios, as the LLM demonstrated poor performance on more intricate decision-making tasks, with its reported confidence often failing to align with its actual success in these cases. The findings underscore the need for clearer deprescribing guidelines, improved LLM calibration for real-world use, and better integration of human–artificial intelligence workflows to balance artificial intelligence recommendations with clinician judgment.

## Introduction

Polypharmacy, widely defined as the regular use of at least 5 medications, is common in older adults and at-risk populations [1]. In fact, approximately 30% of patients aged 65 years or older have polypharmacy [2], and nearly half of older emergency department (ED) patients are discharged with one or more new medications [3]. Although necessary and beneficial for some patients, polypharmacy can increase the risk of negative consequences for patients, including ED visits, adverse drug events, falls, disability, and inappropriate medication use [1]. While definitions differ, deprescribing is generally defined as a structured process by which potentially inappropriate medications (PIMs) are identified and withdrawn under the supervision of a health care provider. In some definitions, the process is described as evaluating the risk-benefit tradeoff, focusing on situations where the potential or actual harms of a medication outweigh its benefits, considering the patient’s individual care goals and quality of life [2,4,5].

Deprescribing tools, such as the Screening Tool of Older People’s Prescriptions (STOPP) [6] and Beers criteria [7], have been developed to help providers assess and identify PIMs based on a patient’s medication list [7-9]. These explicit assessments are criterion-based with clear standards but are often impractical to implement in time-constrained clinical settings, such as ED, due to the need to evaluate multiple clinical indications and specialist-prescribed medications [10]. Attempts to digitize these criteria into electronic clinical decision support (CDS) have raised difficulties, typically requiring a labor-intensive coding process and unstructured information such as free text from patient records to contextualize certain criteria [11,12].

Large language models (LLMs) have been shown to interpret complex clinical situations and offer recommendations, from differential diagnoses to care management, leading to growing interest in their application in the medical field [13-16]. Moreover, they have been shown to extract medication-related data such as medication name, dosage, and frequency, necessary for the application of deprescribing criteria [17]. Finally, LLMs are excellent in-context learners, requiring very little labeled data to make predictions [18]. This reduces the annotation burden for time-constrained ED physicians while improving the use of unstructured patient records to contextualize patient medication lists. However, the majority of clinical reasoning evaluations on LLMs have been conducted using standardized exams (the United States Medical Licensing Examination) or digital case reports [14,19]. Their ability to perform clinical reasoning and calibrate responses over physician-generated text remains understudied.

Here, we propose to evaluate the performance of an LLM-based data pipeline in recommending deprescribing options for older adult ED patients at discharge based on 2 leading deprescribing criteria, Beers and STOPP. We have also included a recently developed list of criteria, Geriatric Emergency Medication Safety Recommendations (GEMS-Rx), intended to prevent the initiation of inappropriate medications in the acute care setting, as similar deprescribing lists specific to this care environment are not available [3]. Through this work, we hope to evaluate whether an LLM-based CDS system can effectively triage medications eligible for electronic deprescribing in older adults. Successful implementation of such a system would help address gaps in electronic deprescribing by using an LLM to contextualize recommendations within individual patient records and reduce manual development in CDS tools.

## Methods

### Ethical Considerations

This study was conducted with approval from the Institutional Review Board (IRB) at Yale University, under protocol number 2000035077. The IRB determined that this research qualifies for exemption as it involves secondary analysis of existing electronic health record (EHR) data, with no additional patient contact or data collection. The original data were collected with patient consent, and the current analysis adheres to the conditions of that consent and IRB approval, permitting secondary analysis without the need for additional consent. All data were de-identified prior to analysis to ensure patient confidentiality. This study complies with ethical standards and guidelines for research involving human subjects.

### Patient Cohort

All older adults (aged 65 years and older) with polypharmacy (5 or more active outpatient medications) presenting to an ED in a large academic medical center in the Northeast United States between January 2022 and March 2022 were identified. Due to budgetary constraints and a lack of prior evidence regarding the performance of LLMs in this task to guide a power calculation, we selected a random sample of 100 unique patients for evaluation.

### Identification of Consensus-Based High-Yield Criteria

We conducted a consensus-based evaluation to filter three preexisting deprescribing lists (ie, STOPP [6], Beers [7], and GEMS-Rx [3]) into a focused set of high-yield deprescribing criteria for the LLM to use in its recommendations. High-yield criteria were defined as those posing a significant clinical risk to the patient and being identifiable within the electronic health records (EHRs). To identify these criteria, we evaluated 180 recommendations across 2 key dimensions: clinical risk and EHR computability. The consensus panel consisted of 6 board-certified physicians (in Emergency Medicine, Internal Medicine, and Clinical Informatics) and 1 ED pharmacist. Each member of the group individually reviewed each of the criteria and rated them on a 5-point Likert scale. We selected the top 50% of criteria with an average score greater than 3 on both dimensions, calculated across all experts, as high-yield criteria. We further elaborate on this consensus process and the final set of criteria in the Results section.

The need to filter the criteria before proceeding with the study was identified in our preliminary research [20], which revealed that one of the main causes of discrepancies between physicians and LLMs arose from ambiguous inclusion or exclusion conditions in deprescribing criteria. For example, criteria like “Statins for primary cardiovascular prevention in persons aged ≥85 with established frailty with expected life expectancy likely less than 3 years” include elements—such as “established frailty” and “expected life expectancy”—that are challenging to quantify and therefore difficult to implement computationally. The dimensions used to filter criteria were chosen to ensure that an LLM-enabled CDS tool prioritizes meaningful recommendations from high-quality EHR data, enabling accurate, actionable deprescribing recommendations and reduction of alert fatigue. We present the final set of high-yield criteria based on the average results of the consensus study.

### Deprescribing Recommendations by GPT-4o

**Figure 1:**
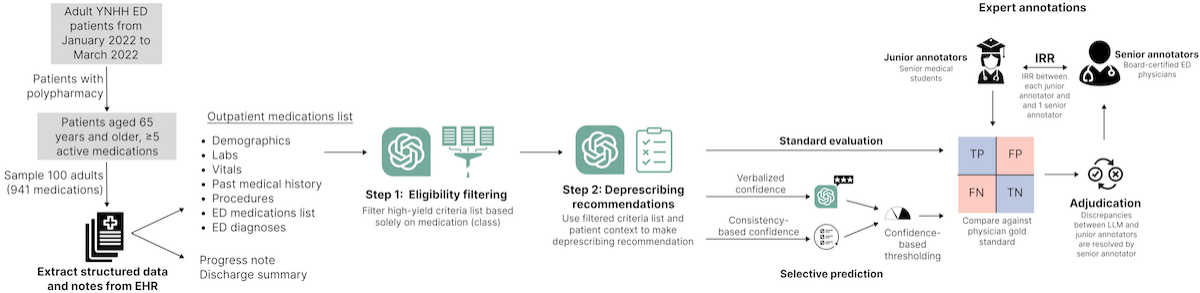
Overview of the evaluation pipeline, consisting of a 2-step GPT-4o process, performance comparison with junior annotators (medical students), and final adjudication by senior annotators (board-certified physicians). ED: emergency department; EHR: electronic health record; FN: false negative; FP: false positive; IRR: interrater reliability; TN: true negative; TP: true positive; YNHH: Yale New Haven Hospital.

The study was approved under an exemption by the Yale University institutional review board prior to commencement (HIC# 2000035077). All patient-level data were deidentified prior to use with the LLM. We leveraged Microsoft’s Azure OpenAI GPT-4o (GPT-4o model version: 2024-08-06 and OpenAI API version: 2024-02-15-preview) to produce deprescribing recommendations through a 2-stage process, as shown in Figure 1. In stage 1, GPT-4o was prompted to filter the full list of high-yield criteria solely based on the patient’s medication list, ignoring inclusion or exclusion conditions. In stage 2, GPT-4o was prompted to use its previously filtered criteria list, along with structured (eg, demographics, lab values, vitals, and past medical history) and unstructured (most recent progress note and discharge summary) information, to determine if the patient satisfied any deprescribing criteria and the medication should be recommended for deprescribing. Each medication was evaluated individually to prevent errors from simultaneous processing, such as misattribution of criteria or medication omissions. To ensure optimal performance, we engineered prompts in an iterative fashion [21], using 1 patient at a time from a set of patients (up to 10% of the cohort) not used in the subsequent evaluation. After each evaluation, prompts were adjusted to correct any systematic errors (eg, instances where no relevant criteria in step 1 led to noncriteria-based deprescribing recommendations in step 2) by the LLM. After our third prompt yielded an output without any identifiable errors, we stopped the iterative prompt development process. Consequently, the final 2 patients initially reserved for this purpose were included in the final cohort evaluation (n=92 patients, 626 medications). Aside from the consistency-based method described later, all LLM calls were performed with a fixed temperature (temperature=0; low randomness in generated responses) and seed to ensure reproducibility and deterministic outputs.

This 2-stage process was developed to correct errors identified when both stages were accomplished at once. Our initial testing revealed that providing the LLM with the full set of criteria and medications led to simple reasoning errors. The large number of criteria, combined with the simultaneous processing of the complete patient medication list, resulted in inaccuracies in applying individual criteria to specific medications. Separating the process of criteria filtering and application both reduces confusion due to large input context sizes [22] and ensures that extraneous context does not distract the LLM [23]. The full prompts for step 1 and step 2 are included in Figures S5 and S6 in Multimedia Appendix 1.

### Selective Prediction Methods

In addition to evaluating an LLM’s ability to make deprescribing recommendations, we assessed whether its confidence estimates were well-calibrated and examined their impact on predictive performance. To do so, we collected GPT-4o’s decision confidence for both steps using 2 validated confidence elicitation methods: chain-of-thought verbalized confidence and self-random sampling with average-confidence aggregation, referred to as consistency-based confidence [24]. In verbalized confidence, we asked the LLM to explicitly estimate its confidence for each step following its decision. For the consistency-based approach, we followed the best practices established in prior work [24] by sampling the LLM multiple times (number of samples=5) with high temperature (T=0.8; high randomness in generated responses) and used a majority vote weighted by the confidence of each response to determine the final deprescribing recommendation.

In a human-in-the-loop decision-making system, the LLM’s confidence would be used to determine if the model should abstain due to low certainty regarding its own decision. In practice, this case would be considered too difficult for the LLM and forwarded to an expert reviewer. This human-in-the-loop decision-making pipeline is known as selective prediction and has been commonly found to improve performance in non–text-based applications [25,26]. We evaluated both selective prediction methods using risk-coverage curves [27,28], substituting risk for the *F*_1_-score (a measure of the predictive performance of a model balancing precision and recall) to capture the full range of predictive performance. Coverage was also expressed inversely as the deferring fraction, representing the proportion of instances where the LLM abstained from making a decision. We conducted a more in-depth analysis of the method that proved to be more effective.

### Comparison and Adjudication With Clinical Experts

In this study, we used a rigorous human review and adjudication process to assess model performance. Two trained senior medical students (M4) classified all medications in the test cohort using a 2-stage pipeline, after first computing interrater reliability (IRR) on an adjudication set of 75 medications from 5 patients. Similar to the LLM pipeline, for each medication, a medical student determined (1) if there exists a relevant high-yield criteria based on the medication list, and (2) whether the medication should be recommended for deprescribing. Discrepancies between the students (junior annotators) and the LLM across both stages were adjudicated by 2 board-certified ED physicians (senior annotators). Similarly to the junior annotators, we measured the IRR between the 2 senior ED physicians, prior to adjudication on the full set of discrepancies. Finally, we classified the errors leading to incorrect recommendations by the LLM, leveraging a prior evaluation framework [29]. We classified each error as 1 of 4 error types: incorrect reading comprehension, incorrect recall of knowledge, incorrect reasoning step, and not enough information, as described in Table 1.

**Table 1:** Definitions of GPT-4o error types inspired by framework from Lièven et al [29] relevant to deprescribing recommendations.

GPT-4o error types	Definition	Example
Incorrect reading comprehension	Includes misunderstanding of order of text, such as when a medication is dependent on another medication in a specific arrangement. Also includes ignoring information provided in the input text, such as missing a relevant category explicitly stated in the recommendations.	GPT failed to recognize acetaminophen by name from the list of STOPP^a^ criteria in a patient at risk for malnutrition or liver disease.
Incorrect recall of knowledge	Includes failure to recognize classes of medications or other medical facts necessary to perform the task.	GPT correctly recognized amlodipine was a calcium channel blocker but failed to recognize it was more broadly an antihypertensive.
Incorrect reasoning step	Faulty reasoning, such as inappropriate assumptions or leads of logic unsupported by the clinical data.	GPT recommended discontinuing warfarin in a patient with a therapeutically elevated INR^b^ after assuming that this elevated INR was due to a bleeding disorder.
Not enough information	Inappropriate application of missing data leading to potentially unreliable conclusions, such as assuming abnormality of a missing laboratory study.	GPT recommended discontinuing a QT^c^ prolonging antidepressant based on the possibility of QT prolongation without any ECG^d^ data or history of abnormal QT interval.

^a^STOPP: Screening Tool of Older People’s Prescriptions.

^b^INR: International normalized ratio.

^c^QT: QT interval.

^d^ECG: electrocardiogram.

### Data Analysis

We evaluated whether the LLM or the medical student was correct, using a gold standard derived from senior annotator (board-certified ED physicians) adjudication of discrepancies. To compare their proportions of correct responses, we applied the McNemar test, a statistical method commonly used to analyze paired nominal data, such as diagnostic accuracy from different assessments applied to the same cases [30]. All analysis was performed using Python (version 3.9; Python Software Foundation). Statistical testing was carried out using *statsmodels* (version 0.14.4) [31] and all visualizations were generated using *seaborn* (version 0.13.2) [32] and *matplotlib* (version 3.8.2) [33].

## Results

### Patient Cohort

**Figure 2:**
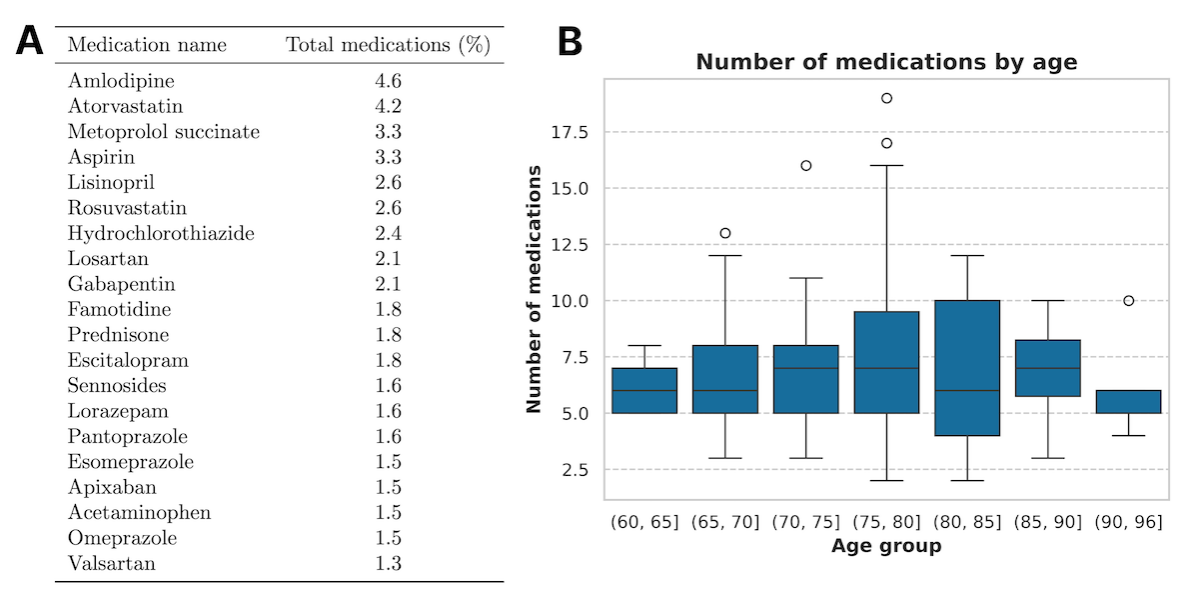
Medication information about baseline cohort. (A) The top 20 most commonly prescribed medications represented as a percentage of the total medication set. (B) Distribution of medications across different age groups.

In total, we identified 10,977 unique patients across 15,161 emergency department encounters from January 2022 to March 2022 meeting our selection criteria, from which 100 patients were randomly selected (Table 2). As our criteria only pertain to oral medication, nonoral medications were subsequently filtered out, resulting in 712 total oral medications across the cohort and a median of 6 oral medications per patient (Figure 2). From our initial study cohort of 100 patients, 10 patients were set aside for both prompt engineering and calculation of IRR between junior annotators. Fewer iterations were needed to refine the prompt than initially anticipated, so the remaining 2 patients were included in the final study cohort. This resulted in a final evaluation cohort of 92 patients, encompassing a total of 626 medications. Based on the mechanism of action, statins were the most common medication class (atorvastatin and rosuvastatin; 6.8% combined), followed by proton pump inhibitors (pantoprazole, esomeprazole, and omeprazole; 4.6% combined). When classified by therapeutic effect, antihypertensive agents were most prevalent (amlodipine, lisinopril, losartan, and valsartan; 10.6% combined).

**Table 2:** Demographic overview of the 100 patients included in the evaluation (N=100).

Characteristics		Values	
Age, mean (SD)		75.8 (7.6)	
**Sex, n (%)**			
	Female		63 (63)
	Male		37 (37)
**Race, n (%)**			
	Asian		1 (1)
	Black or African American		16 (16)
	White or Caucasian		69 (69)
	Other or not listed		13 (13)
	None		1 (1)
**Ethnicity, n (%)**			
	Hispanic or Latino		10 (10)
	Non-Hispanic		90 (90)
**Smoking status, n (%)**			
	Former smoker		47 (47)
	Never smoker		43 (43)
	Current every day smoker		8 (8)
	Passive smoke exposure—never smoker		1 (1)
	Light tobacco smoker		1 (1)
Number of medications, median (IQR)		6.0 (5.0-8.2)	

### Evaluation of Consensus-Based High-Yield Criteria

**Figure 3:**
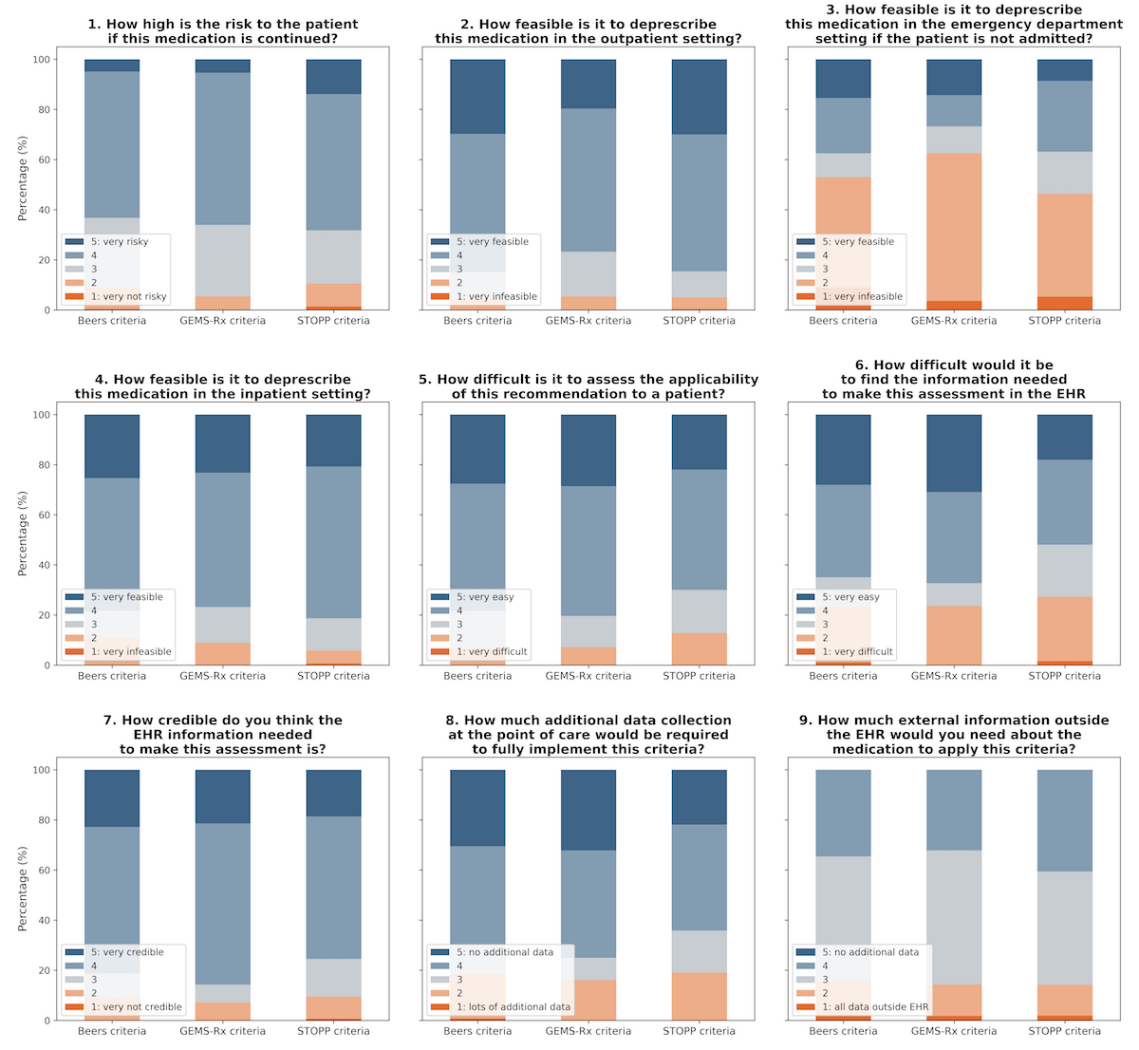
Average distribution of results used to filter high-yield criteria on a 5-point Likert scale from a consensus study by an expert panel (n=7) split by 3 criteria lists: Beers, GEMS-Rx, and STOPP. EHR: electronic health record; GEMS-Rx: Geriatric Emergency Medication Safety Recommendations; STOPP: Screening Tool of Older People’s Prescriptions.

To streamline evaluation by the LLM, we filtered criteria using the average scores from an expert consensus panel. All criteria (n=180) were evaluated based on their scores for clinical risk (Q1) and EHR computability (Q2-Q5), as assessed by an expert panel; average scores by deprescribing list are shown in Figure 3. Any criteria scoring less than 3 on both clinical applicability and EHR computability were excluded resulting in 161 criteria. From the remaining set, the top 50% were selected, resulting in 81 high-yield criteria across all 3 deprescribing lists.

On average, STOPP criteria had the lowest clinical risk and EHR computability ratings, while the Beers criteria had the highest, contributing to their respective adoption rates of 45.9% and 62.5% among high-yield criteria. Reduced inclusion of STOPP criteria was primarily attributed to panelists’ concerns that the information required was not readily accessible within the EHR and would necessitate additional data at the point of care. We also present the results of feasibility in various clinical settings by criteria list in Figure S1 in Multimedia Appendix 1, showing more likelihood for deprescribing in both outpatient and inpatient contexts, compared to the ED, across all 3 criteria lists. A scatter plot of risk versus EHR computability of high-yield criteria is presented in Figure S2 in Multimedia Appendix 1.

### Deprescribing Recommendations by GPT-4o

**Figure 4:**
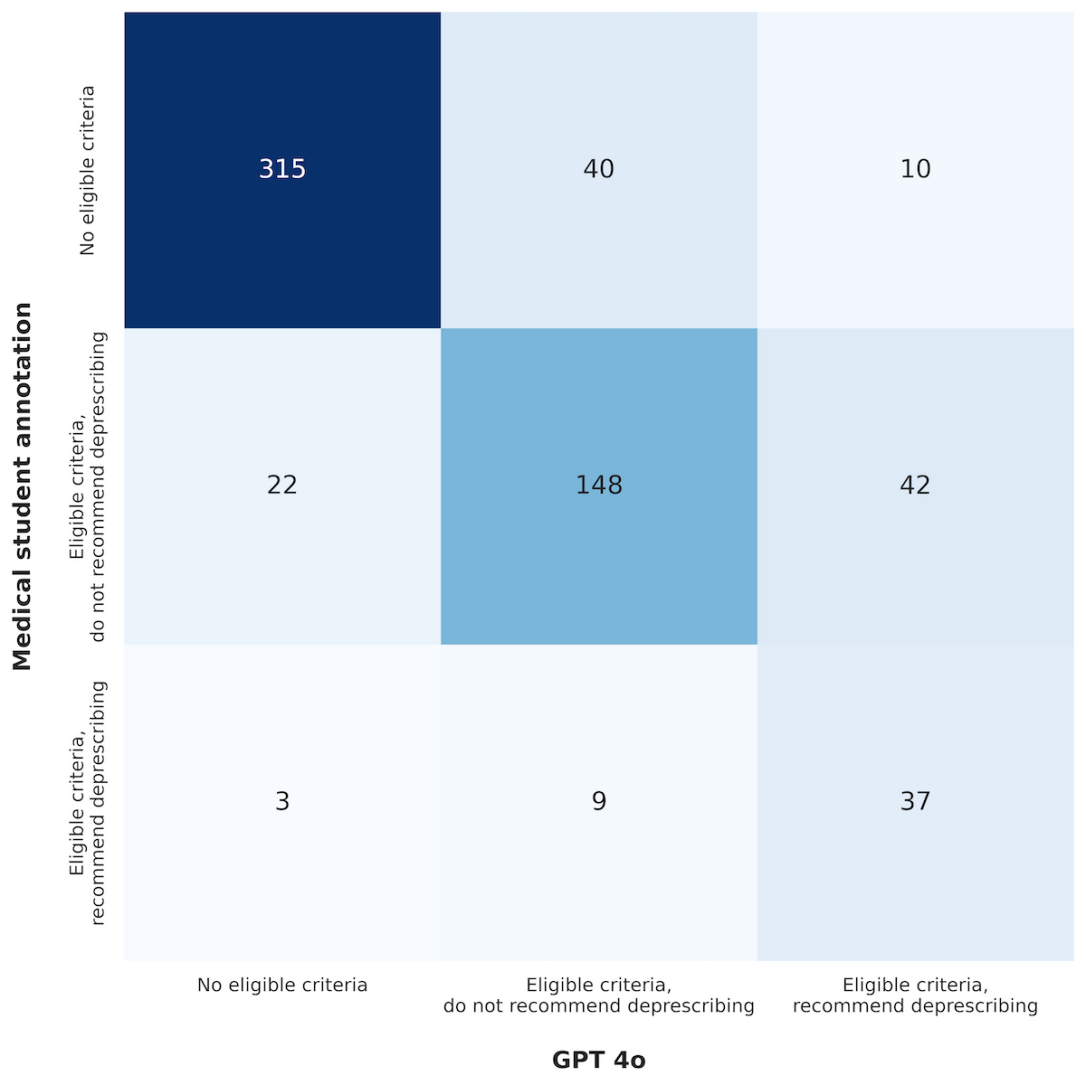
Confusion matrix (n=626 medications). The joint confusion matrix across both steps showing alignment and discrepancies between the GPT-4o model and medical students.

We next evaluated the LLM’s deprescribing recommendations by comparing them to those of medical students, resolving any discrepancies through adjudication by board-certified EM physicians. As shown in Figure 4, 315 medications (50.3% of the total) lacked relevant high-yield deprescribing criteria. The LLM effectively identified these cases, achieving an *F*_1_-score of 0.86 (precision=0.83, recall=0.90). Among those medications with relevant criteria, 64 (10.2% of the total) were cases where either GPT-4o or the medical student recommended deprescribing. In this second step, which involved applying relevant criteria to make a recommendation, the LLM performed less effectively, with an *F*_1_-score of 0.58 (precision=0.47, recall=0.76).

**Figure 5:**
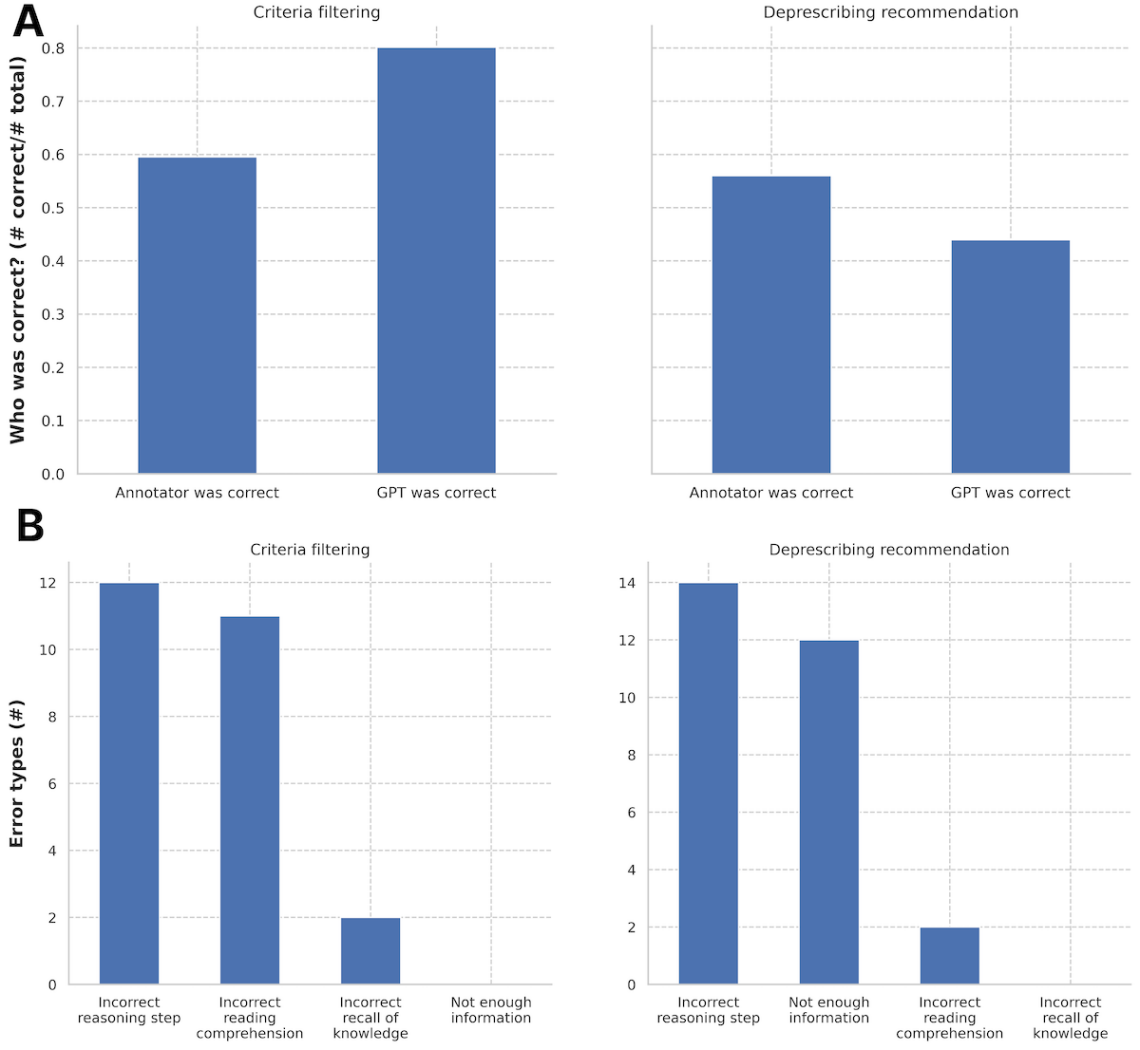
(A) Expert adjudication (n=126). Adjudication by senior clinical expert comparing junior annotators and GPT-4o in both criteria filtering and deprescribing recommendation tasks. (B) GPT-4o error modes. Types of errors by GPT-4o in the adjudication set (n=126) for both criteria filtering and deprescribing recommendation tasks.

For cases where the LLM and the medical students differed, 2 senior annotators (board-certified Emergency Medicine physicians) adjudicated 126 discrepancies after standardizing the codebook and verifying IRR (Cohen *k*: eligibility=0.795, deprescribing=0.745). Notably, the confusion matrix (Figure 4) revealed that a major source of discrepancy was the significantly higher likelihood of the LLM to recommend deprescribing (11.6%) compared to the medical students (1.91%). The confusion matrix describes all possible outcomes when comparing the LLM with the medical students (eg, the medical student recommended deprescribing with eligible criteria and the LLM found no eligible criteria). The adjudication yielded similar results to those observed in comparison with medical students (Figure 5A). Across all discrepancies, GPT-4o was significantly more effective in criteria filtering compared to the medical student (McNemar test for paired proportions [30]: χ^2^_1_=5.985; *P*=.015). However, in applying relevant criteria, GPT-4o performed worse than the medical students, though the difference was not statistically significant (McNemar test: χ^2^_1_=1.818; *P*=.178). Adjudication was chosen as the gold standard for resolving discrepancies because preliminary research showed low IRR among medical students for both steps of the process (Cohen *k*: step 1=0.68, step 2=0.33) across 75 medications.

In cases where GPT-4o was incorrect, error analysis highlighted key patterns across the 2 steps. For criteria filtering, senior annotators observed that errors often stemmed from incorrect reasoning or reading comprehension issues (Figure 5B). Similarly, in making recommendations, the most common error was incorrect reasoning, followed by cases where the LLM lacked sufficient information to make an accurate determination and subsequently made inappropriate assumptions.

### Selective Prediction Methods

**Figure 6:**
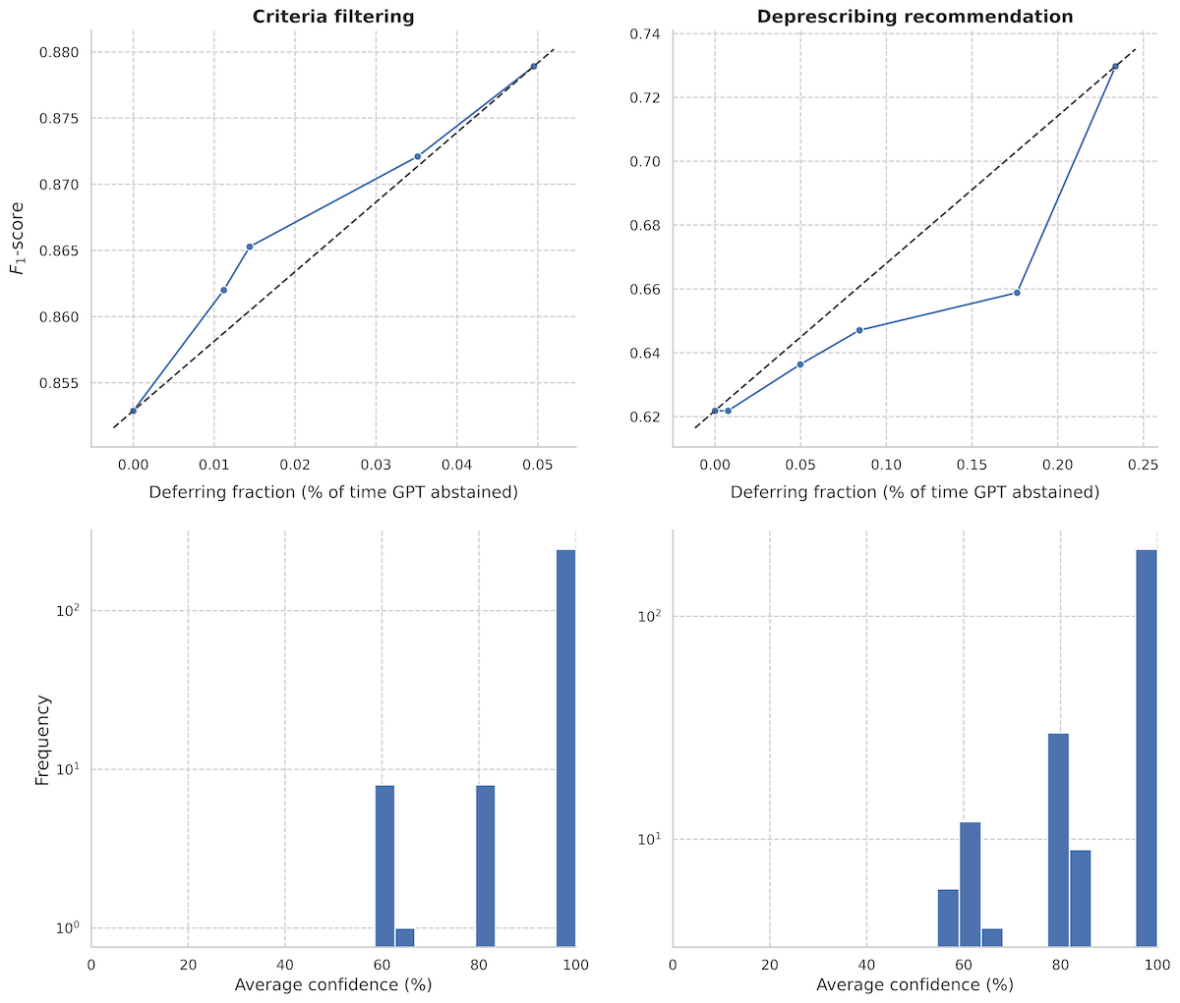
Consistency-based selective prediction. (A) Range of F1-scores resulting from the application of consistency-based selective prediction in both steps of the deprescribing pipeline. The dotted line shows ideal performance as a function of deferring fraction. (B) Distribution of confidences for deprescribing recommendations from GPT-4o on a log scale.

Finally, we investigated the impact of incorporating confidence estimates from the LLM to guide selective prediction, allowing the model to abstain from making predictions in cases of low confidence. We compared 2 approaches: verbalized confidence and consistency-based confidence. Verbalized confidence demonstrated a narrower range of *F*_1_-scores overall, with step 1 (eligibility) ranging from 0.860 to 0.863 and step 2 (deprescribing) from 0.58 to 0.69, as shown in Figure S3 in Multimedia Appendix 1. In contrast, consistency-based confidence exhibited broader and higher *F*_1_-score ranges, with step 1 spanning 0.85 to 0.88 and step 2 ranging from 0.62 to 0.73 (Figure 6A). These results suggest that consistency-based confidence provides more flexibility and improved performance compared to verbalized confidence across both steps.

In particular, consistency-based selective prediction demonstrates a positive linear relationship between accuracy in deprescribing recommendations and deferring fractions. However, despite some minor improvements, we find that the LLM is poorly calibrated, as shown in Figure 6B. Despite consistency-based weighting, the confidence distribution is severely left-skewed with the minimum confidence being 58.5% in eligibility filtering and 54.5% in deprescribing recommendations.

## Discussion

### Principal Findings

In this retrospective cohort study evaluating deprescribing opportunities for PIMs among older adults with polypharmacy in the ED, we found that LLMs effectively identify relevant criteria from verified lists but are less adept at applying these criteria to individual patient cases. GPT-4o’s performance was compared to that of medical students in a 2-step pipeline: filtering for criteria-eligible medications and making specific deprescribing recommendations. Adjudication by senior clinicians was used to resolve discrepancies, and selective prediction methods were tested to improve the model’s reliability. The results offer insights into both the capabilities and limitations of LLMs in a real-world clinical context, highlighting key areas for improvement in both LLM frameworks and deprescribing guidelines.

### Effectiveness of the 2-Step LLM Pipeline

The LLM demonstrated strengths in the initial filtering step, accurately identifying a high proportion of medications that matched deprescribing criteria, thus offering the potential to support clinicians in rapidly screening complex medication lists. In fact, the LLM outperformed medical students by a significant margin (80.1% vs 59.5% correct, McNemar test: *P*=.02). The adjudication, combined with strong overall performance (maximum *F*_1_-score: 87.8%) using selective prediction methods, suggests that the LLM can effectively minimize the number of criteria requiring final review for deprescribing recommendations. Cases of misclassification were relatively uncommon and primarily related to nonstandard drug class names or overly broad groups, which could be improved with refined deprescribing criteria. However, in the second step—making specific deprescribing recommendations—the LLM encountered considerable difficulty, particularly when dealing with ambiguous criteria, missing information, and nuanced clinical scenarios. For example, thiazide diuretics are recommended for deprescribing in cases of significant hypokalemia, hyponatremia, hypercalcemia, or a history of gout. However, GPT-4o recommended deprescribing without access to current electrolyte values, instead basing its suggestion on a history of chronic kidney disease, a condition associated with potential electrolyte imbalances but not meeting the relevant inclusion criteria. If implemented in clinical decision support, these inaccuracies might contribute to increased alert fatigue and extend the time required to interpret LLM-generated recommendations, potentially offsetting the intended efficiency gains in identifying deprescribing opportunities.

Our findings on the LLM’s performance in identifying medications with relevant deprescribing criteria based on eligibility guidelines align with evidence from clinical trial matching literature [34], where LLMs have shown performance comparable to physicians in applying such criteria to identify eligible patients. One study used a similar “filter-and-apply” pipeline, in which trials were first filtered and then matched to patients, showcasing the effectiveness of this approach [35]. However, despite successes in eligibility filtering, challenges remain when applying complex criteria to specific patient cases. Similar errors to those observed in our work have been reported, such as incorrectly identifying patients who meet partial criteria, or assuming a patient with breast cancer does not have lung cancer simply because it is not explicitly mentioned [36,37]. Overall, while LLMs hold promise for reducing the time burden in determining deprescribing eligibility, their application requires careful consideration, particularly in tasks involving complex clinical reasoning.

#### Role of Selective Prediction in Clinical Decision-Making

To address the model’s limitations in clinical decision-making, we implemented selective prediction methods, which allowed the LLM to “abstain” from making a recommendation in cases of low confidence. Selective prediction marginally improved the LLM’s filtering accuracy by enabling it to defer uncertain cases to human reviewers. However, the effectiveness of this approach was limited by the poorly calibrated confidence levels assigned by the LLM to its decisions. Specifically, the LLM displayed a minimum confidence level of 54%, even in cases where its recommendations were incorrect. This indicates a tendency toward overconfidence, particularly in its deprescribing recommendations. While verbalized confidence is known to be overconfident in clinical question answering [38], our results contradict recent work that suggests that consistency-based methods alleviate some of these concerns [39]. This discrepancy underscores the importance of task-specific confidence thresholds and suggests that selective prediction, while useful, is not a one-size-fits-all solution in complex clinical applications.

Improved uncertainty calibration in LLMs could enhance selective prediction methods, optimizing physician-artificial intelligence (AI) workflows in clinical settings. Future applications of a well-calibrated deprescribing CDS tool could flag cases where critical information is missing (eg, antipsychotics at unchanged doses for more than 3 months without a documented medication review). This approach could streamline medication filtering while preserving human oversight, allowing clinicians to focus on complex cases where LLM reliability is limited.

#### Need for Clearer Deprescribing Guidelines

A notable finding from this study is the need for clearer and more consistent deprescribing guidelines. Ambiguities in criteria definitions, such as those related to medication administration routes and drug classes, present substantial barriers to automation and contribute to discrepancies between human and model interpretations. Additionally, the model often recommended deprescribing medications that, while potentially inappropriate, required specific contextual qualifiers (eg, patient’s life expectancy, nutritional status, frailty status) to justify deprescribing—criteria that the LLM misapplied due to lack of explicit context or ambiguous language in the guidelines. It is important to note that current deprescribing criteria were not originally designed for direct implementation in CDS systems, but rather as general recommendations for prescribing physicians. Reorganizing these criteria into a structured, explicit framework tailored for CDS use could reduce ambiguity, improve the model’s performance, and support more consistent application in clinical practice. In general, streamlining deprescribing criteria to ensure consistent application across clinical contexts could improve model reliability and help standardize deprescribing practices.

#### Implications for LLM Use in Clinical Practice and Future Directions

The results of this study underscore the promise of LLMs in enhancing deprescribing workflows by providing rapid filtering of PIMs, which could alleviate some of the burdens on health care providers. However, this work also highlights the limitations of current LLMs in complex, context-sensitive clinical decision-making tasks. The LLM’s frequent tendency to overrecommend deprescribing, as compared to medical students, indicates that clear boundaries for medication eligibility and exclusion are critical for reducing false positives in automated recommendations. Cases with the potential for human harm were observed, such as suggesting deprescribing anticoagulation in a patient with recent thromboembolism. Additionally, cases were seen in which the LLM recommended deprescribing without citing a specific criterion. These behaviors suggest that strong guardrails on the LLM are needed to ensure safe, high-quality recommendations. Enhancing guideline specificity, particularly for complex inclusion or exclusion criteria, could reduce both human and model error rates and may foster greater acceptance of AI-assisted deprescribing tools among clinicians. Our findings highlight the potential value of human-AI collaboration frameworks. For example, a human-in-the-loop framework (a model in which humans review difficult cases the LLM cannot resolve) could involve LLMs assisting in the identification of deprescribing opportunities while deferring final recommendations to clinicians [40]. Alternatively, the LLM could focus on the initial filtering of relevant deprescribing criteria for specific medications, leaving the recommendation task entirely to the clinician, thereby leveraging the LLM’s strength in mapping medications or medication classes to appropriate criteria efficiently [41]. These approaches not only leverage the model’s efficiency in data processing but also mitigate risks associated with erroneous recommendations, particularly in this high-risk clinical context. Future research should focus on refining LLM architectures to better handle the nuances of clinical reasoning and context interpretation, perhaps by incorporating more advanced natural language processing techniques and domain-specific training. Additionally, efforts to standardize deprescribing guidelines would greatly benefit the development of automated tools in this area, making them more reliable and broadly applicable.

### Limitations

This study has several limitations that warrant consideration. First, the retrospective nature of our analysis, relying on historical data from EHRs, may not fully capture the complexity of real-time clinical decision-making in emergency settings. The study’s focus on a single large academic medical center limits the generalizability of our findings to other settings with different patient populations, documentation patterns, and health care practices. Second, the selective prediction methods, while providing insights into the LLM’s confidence, were not universally effective, particularly in the nuanced task of deprescribing recommendations. The model’s performance in these recommendations highlights the challenge of translating structured criteria into actionable clinical decisions, especially when faced with ambiguous inclusion or exclusion conditions. Additionally, the model’s reliance on textual prompts and structured EHR data may not fully account for nuanced clinical contexts that influence deprescribing decisions. Third, the small sample size for detailed analysis (100 patients) limits the statistical power and may not reflect broader patterns of medication use and deprescribing needs. Cost may be a barrier to larger sample sizes in the future, as the total application programming interface utilization fees were approximately US $300-$400 over these 100 patients and the cost to both evaluate and implement the system in the real world would scale linearly with the study population. Additionally, the study relied on medical students for initial annotation. While these annotations were reviewed and adjudicated by board-certified physicians, this process may introduce variability, potentially affecting the reliability of their use as a gold standard in selective prediction methods. Our process for selecting these criteria also did not explicitly include any geriatricians, though did include a range of individuals who regularly care for older adults. Finally, the criteria used (STOPP, Beers, and GEMS-Rx) were selected based on their perceived clinical risk and EHR computability, which may not encompass all relevant deprescribing scenarios. The lack of standardized guidelines for implementing deprescribing criteria in LLMs also poses a challenge to consistency and accuracy.

### Conclusions

This study demonstrates the potential of LLMs to augment clinical decision support by effectively filtering deprescribing criteria for older adults with polypharmacy in ED. While the LLM showed promise in identifying medications eligible for deprescribing, it faced challenges in making nuanced deprescribing recommendations, underscoring the need for human oversight in AI-driven processes. Future research should prioritize refining the model by addressing ambiguities in deprescribing criteria and integrating broader clinical context, such as longitudinal data from prior progress notes and discharge summaries, to enable the detection of relevant clinical trends. Expanding the dataset and exploring more effective strategies for integrating human judgment with AI capabilities will help overcome limitations in generalizability, helping optimize patient care. The findings underscore the potential of LLMs in AI-enabled automated CDS tools for deprescribing while emphasizing the need to refine deprescribing criteria and establish clearer guidelines to support the integration of AI into clinical practice.

## Appendix

Multimedia Appendix 1 Additional figures and tables.

## Abbreviations

AI: artificial intelligence
CDS: clinical decision support
ED: emergency department
EHR: electronic health record
GEMS-Rx: Geriatric Emergency Medication Safety Recommendations
IRR: interrater reliability
LLM: large language model
PIM: potentially inappropriate medication
STOPP: Screening Tool of Older People’s Prescriptions
