# Supplementary material for: Identifying Deprescribing Opportunities With Large Language Models in Older Adults: Retrospective Cohort Study
Source: JMIR Aging. 2025 Apr 11;8:e69504. doi: 10.2196/69504 (PMC12032504; doi:10.2196/69504)
Supplement: Multimedia Appendix 1 [file aging_v8i1e69504_app1.docx]

**Multimedia Appendix** for “Identifying Deprescribing Opportunities with Large Language Models in Older Adults” in JMIR Aging

**
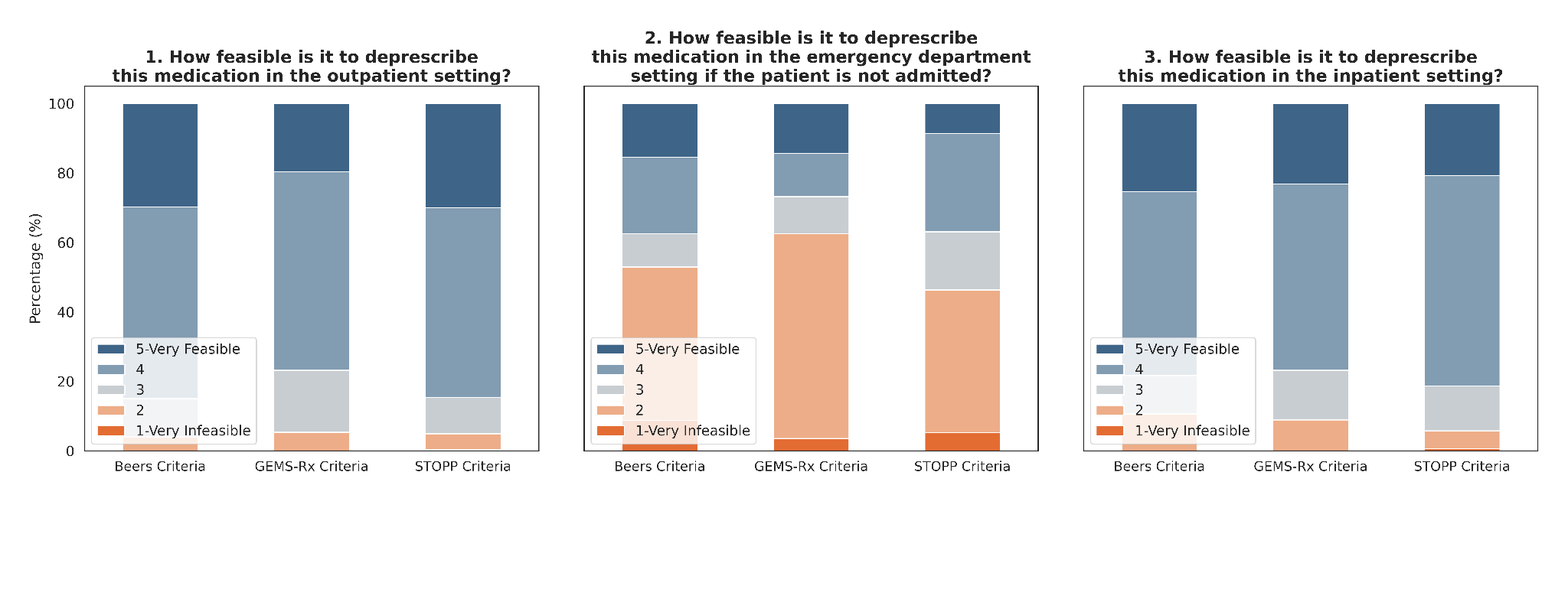
**

**Figure A1.** Average distribution of results of feasibility questions on 5-point Likert scale from consensus study by expert panel (n=7) split by three criteria lists: Beers, GEMS-Rx, and STOPP.


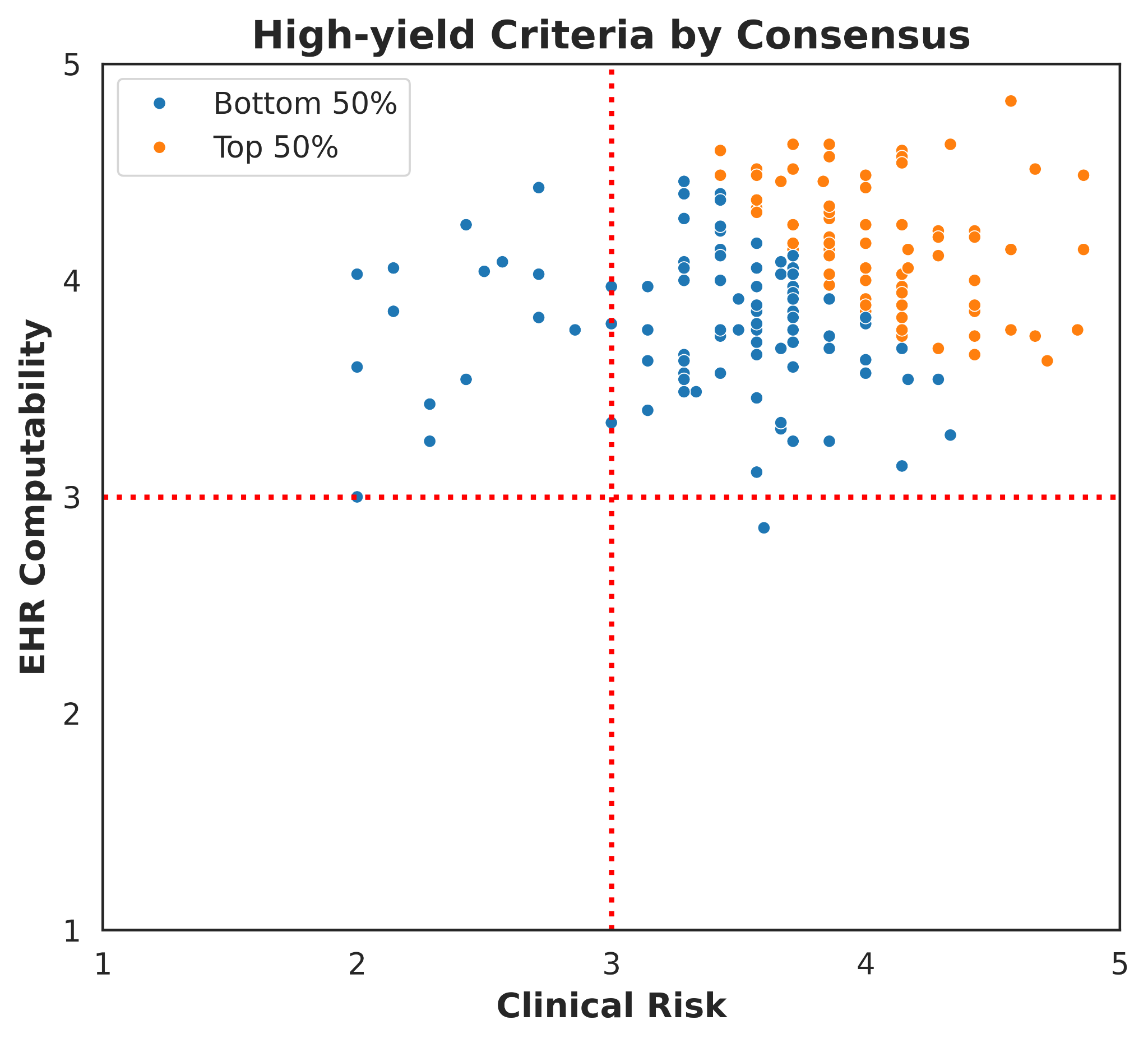


**Figure A2.** Selected deprescribing criteria as a function of patient risk and Electronic Health Record (EHR) computability from the consensus-based evaluation of Screening Tool of Older People’s Prescriptions (STOPP), Beers, and GEMS-Rx. Red dotted lines indicate cutoffs at clinical risk >3 and EHR computability >3. Given the large number of criteria still within the upper right quadrant (potentially high yield), the top 50% of the quadrant was selected (orange) as the final list.


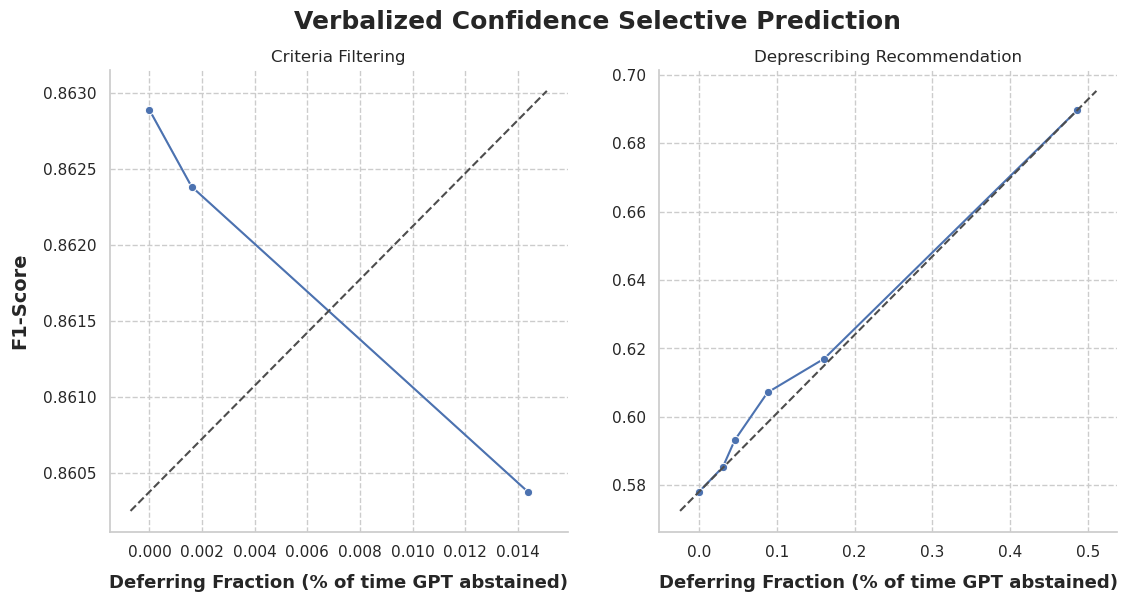


**Figure A3.** Range of F1-scores resulting from the application of verbalized confidence-based selective prediction for both steps of the deprescribing pipeline. The dotted line shows ideal performance as a function of deferring fraction.


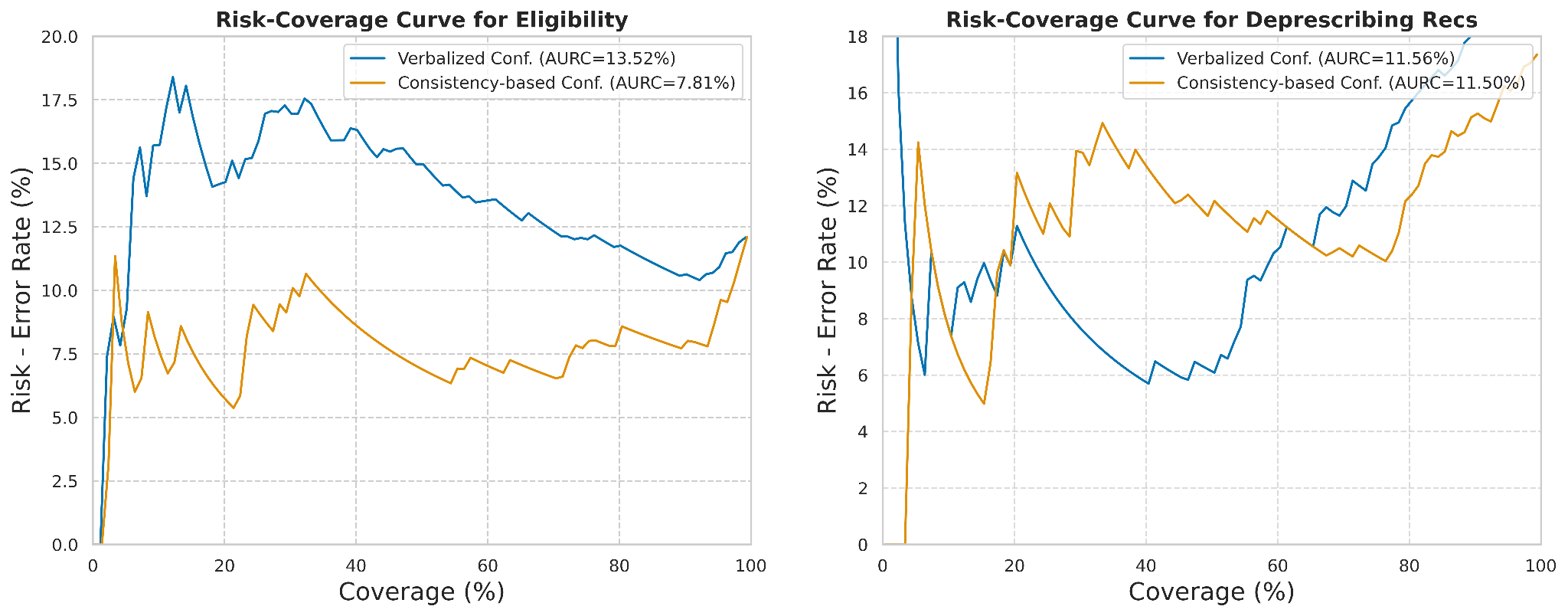


**Figure A3.** Risk-coverage plots for Step 1 (eligibility filtering) and 2 (deprescribing recommendations) for two methods of confidence elicitation: verbalized and consistency-based using avg.-confidence aggregation. AURC = area under the risk-coverage curve. AURC closest to 0 represents better performance as error rates regardless of model coverage should be low.

**
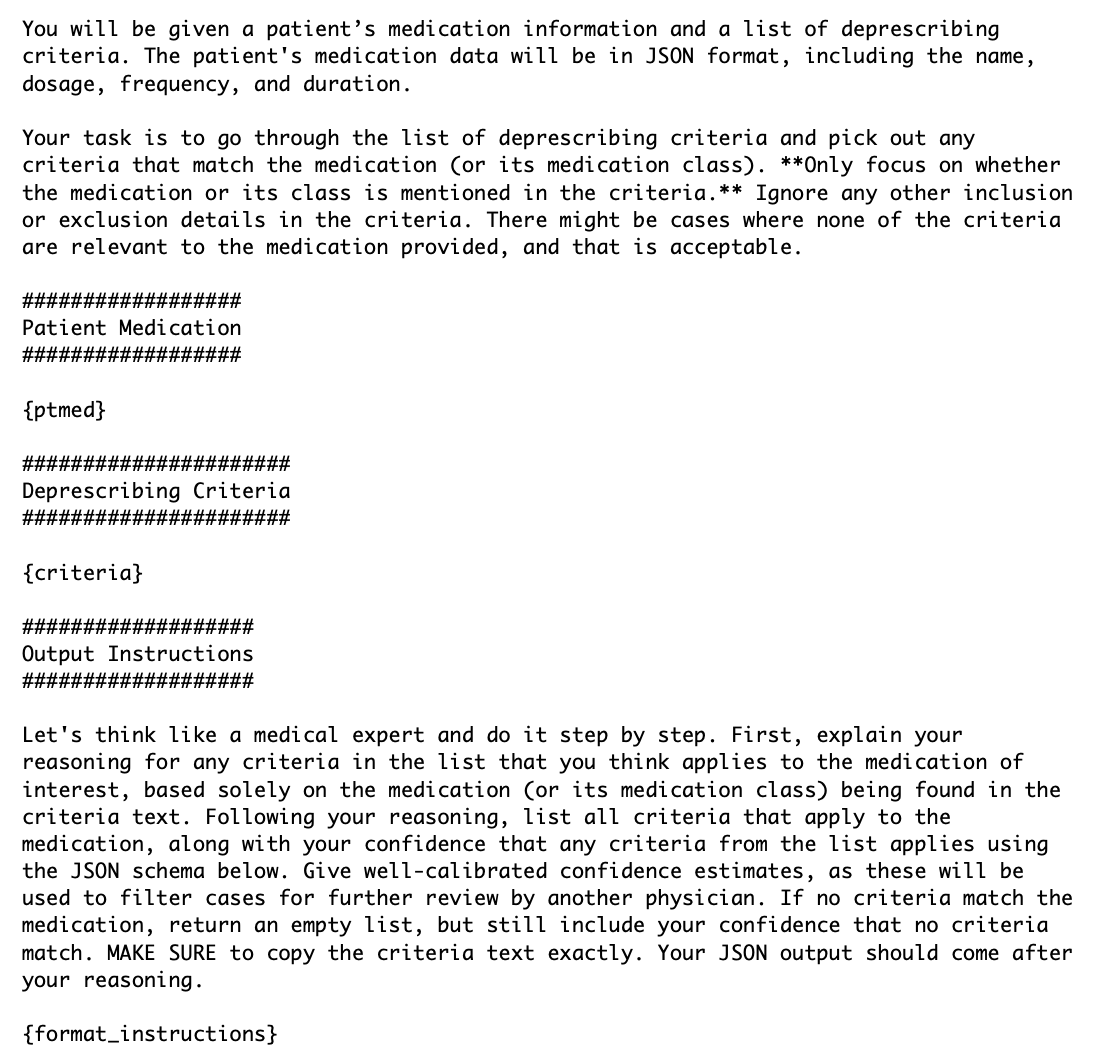
**

**Figure A5.** Prompt for Step 1 (Eligibility Filtering). ptmed is a single med from a patient’s outpatient medication list, criteria is the full list of high-yield criteria to be filtered, and format_instructions are the instructions to output the relevant, filtered criteria and confidence in a structured JSON format.


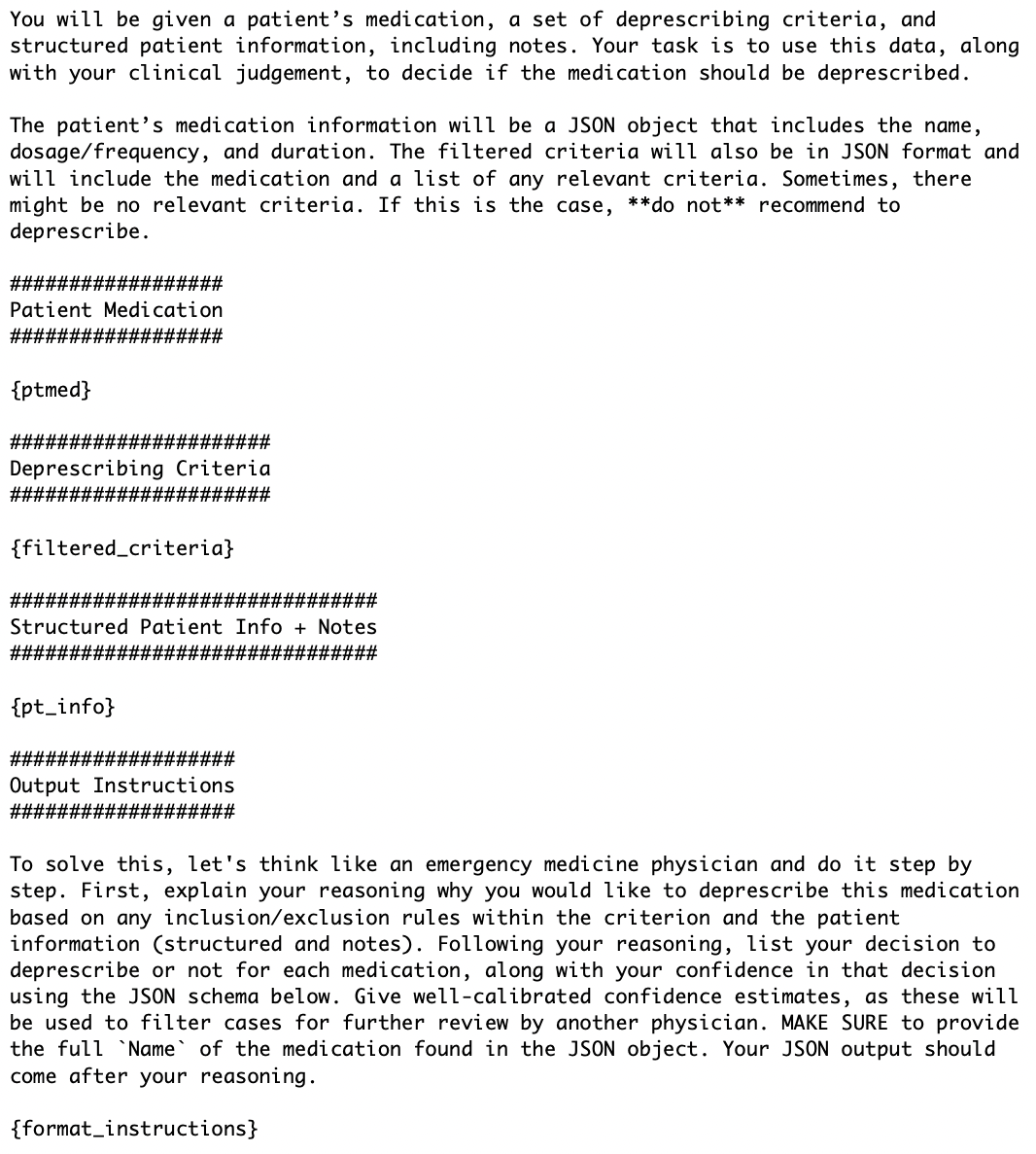


**Figure A6.** Prompt for Step 2 (Deprescribing Recommendations). ptmed is a single med from a patient’s outpatient medication list, filtered_criteria is the list of filtered high-yield criteria from Step 1, pt_info is the formatted text of the structured patient information and most recent progress note and discharge summary with delimiters, and format_instructions are the instructions to output the deprescribing recommendation and confidence in a structured JSON format.

**Table A1.** List of high-yield criteria by deprescribing list

| **Beers Criteria** | American Geriatrics Society 2023 updated AGS Beers Criteria® for potentially inappropriate medication use in older adults. J Am Geriatr Soc. 2023;71(7):2052-2081. doi:10.1111/jgs.18372  Reference the original published version for the full text of recommendations which were not available in the public domain.  Table 2: Rows 1, 2, 6, 8, 9, 16, 17, 18, 19, 20, 21, 23, 25, 34, 35, 36 |
| --- | --- |
| **GEMS-Rx** | Skains RM, Koehl JL, Aldeen A, et al. Geriatric Emergency Medication Safety Recommendations (GEMS-Rx): Modified Delphi Development of a High-Risk Prescription List for Older Emergency Department Patients. Ann Emerg Med. Published online March 2024:S0196064424000714.  Reference the original published version for the full text of recommendations which were not available in the public domain.  Table 3: Rows 1, 2, 6, 7 |
| **STOPP Criteria** | This subset of STOPP criteria are reproduced under the Creative Commons Attribution 4.0 International License available at: <https://creativecommons.org/licenses/by/4.0/>  The text of each recommendation was reproduced verbatim.  O’Mahony D, Cherubini A, Guiteras AR, et al. STOPP/START criteria for potentially inappropriate prescribing in older people: version 3. Eur Geriatr Med. 2023;14(4):625-632. doi:10.1007/s41999-023-00777-y  Digoxin for heart failure with normal systolic ventricular function (no clear evidence of benefit)    Ticlopidine in any circumstances (clopidogrel and prasugrel have similar efficacy, stronger evidence and fewer side-effects).    Corticosteroids with a history of peptic ulcer disease or erosive oesophagitis (risk of relapse unless proton pump inhibitor is co-prescribed).    Hypnotic Z-drugs i.e., zopiclone, zolpidem, zaleplon in patients with recurrent falls (may cause protracted daytime sedation, ataxia).    Nitrofurantoin if eGFR < 45 ml/min/1.73m2 (increased risk of nitrofurantoin toxicity).    Megestrol acetate to increase appetite (increased risk of thrombosis and death with unproven efficacy)    Antipsychotics (i.e., other than clozapine or quetiapine) in those with parkinsonism or Dementia with Lewy Bodies (risk of severe extra-pyramidal symptoms).    Angiotensin-Converting Enzyme inhibitors (ACEIs) or Angiotensin Receptor Blockers (ARBs) in patients with hyperkalaemia i.e., serum K > 5.5 mmol/l.    Drugs that predictably prolong the QTc interval (QTc = QT/RR) in patients with known with demonstrable QTc prolongation (to >450 msec in males and >470 msec in females), including quinolones, macrolides, ondansetron, citalopram (doses > 20 mg/day), escitalopram (doses > 10 mg/day), tricyclic antidepressants, lithium, haloperidol, digoxin, class 1A antiarrhythmics, class III antiarrhythmics, tizanidine, phenothiazines, astemizole, mirabegron (risk of life- threatening ventricular arrhythmias).    Z-drugs (zolpidem, zopiclone, zaleplon) for insomnia for ≥ 2 weeks (increased risk of falls, fractures).    Benzodiazepines for agitated behaviour or psychotic symptoms of dementia (no evidence of efficacy).    Centrally-acting antihypertensives e.g., methyldopa, clonidine, moxonidine, rilmenidine, guanfacine (centrally-active antihypertensives are generally less well tolerated by older people than younger people)    NSAID's if eGFR < 50 ml/min/1.73m2 (risk of deterioration in renal function).    Antiplatelet agents with vitamin K antagonist, direct thrombin inhibitor or factor Xa inhibitors in patients with stable coronary, cerebrovascular or peripheral arterial disease (no evidence of added benefit from dual therapy).    Drugs with potent anticholinergics/antimuscarinic effects** in patients with delirium or dementia (risk of exacerbation of cognitive impairment).    Systemic antimuscarinic drugs (e.g., oxybutynin, tolterodine, trospium) with dementia or chronic cognitive impairment (risk of increased confusion, agitation).    Systemic corticosteroids instead of inhaled corticosteroids for maintenance therapy in moderate-severe COPD (unnecessary exposure to long-term side effects of systemic corticosteroids and effective inhaled therapies are available).    Benzodiazepines in patients with recurrent falls (may cause reduced sensorium, impair balance).    Antiplatelet agents in combination with vitamin K antagonist, direct thrombin inhibitor or factor Xa inhibitors in patients with chronic atrial fibrillation, unless there is concurrent coronary artery stent(s) inserted or angiographically proven high grade (> 50%) coronary artery stenosis (no added benefit from antiplatelet agents).    NSAID with concurrent corticosteroids for treatment of arthritis/rheumatism of any kind (increased risk of peptic ulcer disease).    Non-steroidal anti-inflammatory drugs (NSAIDs) other than COX-2 selective agents with history of peptic ulcer disease or gastrointestinal bleeding, unless with concurrent PPI or H2 antagonist (risk of peptic ulcer relapse).    Centrally acting antihypertensives (may impair sensorium and may cause orthostatic hypotension).    Alpha blockers as antihypertensives in patients with recurrent falls (may cause orthostatic hypotension).    Selective serotonin re-uptake inhibitors (SSRI's) with current or recent significant hyponatraemia i.e., serum Na+ < 130 mmol/l (risk of exacerbating or precipitating hyponatraemia).    Benzodiazepines for insomnia for ≥ 2 weeks (high risk of dependency, increased risk of falls, fractures and road traffic accidents).    Systemic oestrogens or androgens with pervious history of venous thromboembolism (increased risk of recurrent venous thromboembolism).    Benzodiazepines with acute or chronic respiratory failure i.e. pO2 < 8.0 kPa ± pCO2 > 6.5 kPa (risk of exacerbation of respiratory failure).    Nonsteroidal anti-inflammatory drugs (NSAIDs) and vitamin K antagonist, direct thrombin inhibitor or factor Xa inhibitors in combination (risk of major gastrointestinal bleeding).    Mineralocorticoid receptor antagonists (e.g. spironolactone, eplerenone) if eGFR < 30 ml/min/1.73m2 (risk of dangerous hyperkalaemia).    Digoxin at a long-term (i.e. more than 90 days) maintenance dose ≥ 125 µg/day if eGFR < 30 ml/min/1.73m2 (risk of digoxin toxicity if plasma levels not measured).    Antiplatelet or anticoagulant drugs with a history of Gastric Antral Vascular Ectasia (GAVE, "watermelon stomach") (risk of major gastrointestinal bleeding).    Acetylcholinesterase inhibitors with concurrent treatment with drugs that induce persistent bradycardia (< 60 beats/min.) such as beta-blockers, digoxin, diltiazem, verapamil (risk of cardiac conduction failure, syncope and injury).    Metformin if eGFR < 30 ml/min/1.73m2 (risk of lactic acidosis).    Factor Xa inhibitors (e.g., rivaroxaban, apixaban, edoxaban) if eGFR < 15 ml/min/1.73m2 (risk of bleeding)    Colchicine if eGFR < 10 ml/min/1.73m2 (risk of colchicine toxicity)    Bisphosphonates if eGFR<30 ml/min/1.73m2 (increased risk of acute renal failure).    Thiazide diuretic with current significant hypokalaemia (i.e., serum K+ < 3.0 mmol/l), hyponatraemia (i.e., serum Na+ < 130 mmol/l) hypercalcaemia (i.e., corrected serum calcium > 2.65 mmol/l) or with a history of gout (hypokalaemia, hyponatraemia, hypercalcaemia and gout can be precipitated by thiazide diuretic)    Phosphodiesterase type-5 inhibitors (e.g., sildenafil, tadalafil, vardenafil) in severe heart failure characterised by hypotension i.e., systolic BP < 90 mmHg, or concurrent nitrate therapy for angina (risk of cardiovascular collapse).    Methotrexate if eGFR <30 ml/min/1.73m2 (increased risk of methotrexate toxicity).    Ventricular rate-limiting drugs i.e., beta blocker, verapamil, diltiazem, digoxin with bradycardia (< 50/min), type II heart block or complete heart block (risk of complete heart block, asystole).    Direct thrombin inhibitors (e.g., dabigatran) if eGFR < 30 ml/min/1.73m2 (risk of bleeding)    Acetylcholinesterase inhibitors with a known history of persistent bradycardia (< 60 beats/min.), heart block or recurrent unexplained syncope (risk of cardiac conduction failure, syncope and injury).    Antihypertensive drugs in severe symptomatic aortic stenosis (risk of severe hypotension, syncope).    Amiodarone as first-line antiarrhythmic therapy in supraventricular tachyarrhythmias (higher risk of major side-effects than beta-blockers, digoxin, verapamil or diltiazem).    Systemic oestrogens with a history of venous thromboembolism (increased risk of recurrence).    Aldosterone antagonists (e.g., spironolactone, eplerenone) with concurrent potassium-conserving drugs (e.g., ACEI's, ARB's, amiloride, triamterene) without monitoring of serum potassium (risk of dangerous hyperkalaemia i.e., > 6.0 mmol/l - serum K should be monitored regularly, i.e., at least every 6 months).    Benzodiazepines for ≥ 4 weeks (no indication for longer treatment; risk of prolonged sedation, confusion, impaired balance, falls, road traffic accidents; all benzodiazepines should be withdrawn gradually if taken for more than 4 weeks as there is a risk of causing a benzodiazepine withdrawal syndrome if stopped abruptly).    Sulphonylureas with a long half-life (e.g., glibenclamide, chlorpropamide, glimepiride) with type 2 diabetes mellitus (risk of prolonged hypoglycaemia).    Concomitant use of two or more drugs with antimuscarinic/anticholinergic properties (e.g., bladder antispasmodics, intestinal antispasmodics, tricyclic antidepressants, first generation antihistamines, antipsychotics) (risk of increased antimuscarinic/anticholinergic toxicity).    Paracetamol at doses ≥ 3 g/24 hours in patients with poor nutritional status i.e., BMI < 18 or chronic liver disease (risk of hepatotoxicity).    Theophylline as monotherapy for COPD (safer, more effective alternative; risk of adverse effects due to narrow therapeutic index).    Systemic oestrogens with a history of breast cancer (increased risk of recurrence).    Verapamil or diltiazem with NYHA Class III or IV heart failure (may worsen heart failure with reduced ejection fraction i.e., HFREF).    Beta-blocker in combination with verapamil.    Memantine with known current or previous seizure disorder (increased risk of seizures).    Antiplatelet agents, vitamin K antagonists, direct thrombin inhibitors or factor Xa inhibitors with concurrent significant risk of major bleeding, i.e. uncontrolled severe hypertension, bleeding diathesis, recent non-trivial spontaneous bleeding (high risk of bleeding).    Any duplicate drug class prescription for daily regular use (as distinct from PRN use) e.g., two concurrent NSAIDs, SSRIs, loop diuretics, ACE inhibitors, anticoagulants, antipsychotics, opioid analgesics (optimisation of monotherapy within a single drug class should be observed prior to considering a new agent).    Systemic oestrogens without progestogens in patients with intact uterus (risk of endometrial cancer).    Use of oral or transdermal strong opioids (morphine, oxycodone, fentanyl, buprenorphine, diamorphine, methadone, tramadol, pethidine, pentazocine) as first line therapy for mild pain (WHO analgesic ladder not observed; paracetamol or NSAID not prescribed as first-line therapy).    Thiazolidenediones (e.g., rosiglitazone, pioglitazone) with heart failure (risk of exacerbation of heart failure).    Corticosteroids (other than periodic intra-articular injections for mono-articular pain) for osteoarthritis (risk of systemic corticosteroid side-effects). |
